# Research Progress on the Preparation of Iron-Manganese Modified Biochar and Its Application in Environmental Remediation

**DOI:** 10.3390/toxics13080618

**Published:** 2025-07-25

**Authors:** Chang Liu, Xiaowei Xu, Anfei He, Yuanzheng Zhang, Ruijie Che, Lu Yang, Jing Wei, Fenghe Wang, Jing Hua, Jiaqi Shi

**Affiliations:** 1 Nanjing Institute of Environmental Sciences, Ministry of Ecology and Environment, Nanjing 210042, China; liuchang@nies.org (C.L.); xuxiaowei@nies.org (X.X.); zhangyuanzheng@nies.org (Y.Z.); yanglu@nies.org (L.Y.); weijing@nies.org (J.W.); 2 School of Chemistry and Chemical Engineering, Nanjing University of Science and Technology, Nanjing 210094, China; cheruijie@njust.edu.cn (R.C.); wangfenghe@njust.edu.cn (F.W.); 3 School of Environmental Science and Engineering, Suzhou University of Science and Technology, Suzhou 215009, China; heanfei@usts.edu.cn

**Keywords:** iron-manganese-modified biochar, environmental remediation, heavy metals, organic pollutants, recycling

## Abstract

Biochar, a porous carbonaceous material derived from the pyrolysis of biomass under oxygen-limited conditions, offers several advantages for environmental remediation, including a high specific surface area, ease of preparation, and abundant raw material sources. However, the application of pristine biochar is limited by its inherent physicochemical shortcomings, such as a lack of active functional groups and limited elemental compositions. To overcome these limitations, metal-modified biochars have garnered increasing attention. In particular, iron-manganese (Fe-Mn) modification significantly enhances the adsorption capacity, redox potential, and microbial activity of biochar, owing to the synergistic interactions between Fe and Mn. Iron-manganese-modified biochar (FM-BC) has demonstrated effective removal of heavy metals, organic matter, phosphate, and nitrate through mechanisms including mesoporous adsorption, redox reactions, complexation, electrostatic interactions, and precipitation. Moreover, FM-BC can improve soil physicochemical properties and support plant growth, highlighting its promising potential for broader environmental application. This review summarizes the preparation methods, environmental remediation mechanisms, and practical applications of FM-BC and discusses future directions in mechanism elucidation, biomass selection, and engineering implementation. Overall, FM-BC, with its tunable properties and multifunctional capabilities, emerges as a promising and efficient material for addressing complex environmental pollution challenges.

## 1. Introduction

Biochar is a porous carbon-based material generated by pyrolysis of biomass under oxygen-limited conditions [1]. Its feedstocks are diverse, primarily derived from agricultural wastes, wood, activated sludge, and animal manure [2,3,4,5]. Owing to its high specific surface area, abundant surface functional groups, and environmentally friendly properties, biochar has been widely applied in areas such as soil remediation, pollutant adsorption, and energy storage [6,7]. However, the performance of unmodified (virgin) biochar is often limited by its inherent characteristics such as a lack of functional groups and limited elemental composition. As a result, its physicochemical properties need to be optimized by means of modification [8,9]. The preparation methods of modified biochar are mainly divided into metal-loaded modification, acid-base modification, oxidant modification, organic solvent modification, mineral modification, plasma modification, and composite modification [10,11,12,13].

In recent years, metal-loaded modified biochar has been extensively applied in the field of environmental pollution remediation, with zero-valent iron (ZVI)-loaded biochar emerging as a prominent variant due to its superior physicochemical properties [1,14,15]. Compared with the original biochar, iron-loaded biochar has significantly improved the adsorption performance, redox capacity, microbial activity, etc., and has shown good ability to remove pollutants in the environment [16,17,18,19,20]. However, the low passivation efficiency of zero-valent iron makes it difficult to form a stable passivation layer in time in the iron-loaded biochar system, and it is easily and rapidly oxidized by environmental substances, reducing the adsorption capacity, selectivity, and stability of the physical and chemical properties of the iron-loaded biochar to ions. This vulnerability substantially reduces the material’s adsorption efficiency, ion selectivity, and physicochemical stability, thereby weakening its adaptability to diverse environmental conditions and limiting its long-term effectiveness. Consequently, these drawbacks restrict the broader application of iron-loaded biochar in environmental remediation [15,21,22].

Compared with single-metal iron-loaded biochar, ferromanganese bimetallic-loaded biochar(FM-BC) demonstrates significantly enhanced pollutant removal efficiency. This improvement is primarily attributed to two key factors. First, the iron-manganese redox synergistic effect is generated, and a dynamic electron transfer chain can be formed between iron and manganese ions, which significantly enhances the redox ability of the material [23]. The strong reducibility of iron (such as the electron release in the process of Fe^2+^→Fe^3+^) can be efficiently captured by the multivalent states of manganese (such as Mn^4+^→Mn^3+^→Mn^2+^), forming a continuous “Fe^2+^→Mn^4+^→pollutant” electron transport chain [24]. This not only prevents Fe^2+^ from being oxidized too quickly by O_2_ in the environment but also amplifies the overall redox efficiency through the valence state cycle of manganese [25]. Second, FM-BC enables the construction of multiple active sites. Manganese oxides provide additional adsorption sites, enhancing pollutant immobilization via coordination mechanisms [26]. Meanwhile, compared with the easy hydrolysis and loss of Fe^3+^ in the single-metal iron system, the introduction of manganese can inhibit the dissolution of iron by forming a Fe-Mn spinel structure with higher chemical stability [27]. In turn, iron can stabilize the high valence state of manganese, reduce the risk of secondary pollution from manganese, and at the same time reduce the leaching of heavy metals and organic matter, collectively extending the service life of the material [28]. Additionally, in catalytic applications, the introduction of Mn also broadens the free-radical generation pathway [29]. Therefore, the Fe-Mn bimetallic loading strategy breaks through the bottleneck of the performance of single-metal Fe-loaded biochar in environmental remediation through electronic coupling and functional complementarity, representing a significant advancement in the development of high-performance materials for environmental remediation [30].

## 2. Preparation Method of Iron-Manganese-Modified Biochar

The preparation process of Fe-Mn-modified biochar (FM-BC) involves a variety of methods, with commonly used techniques including impregnation pyrolysis, hydrothermal synthesis, co-precipitation, sol-gel, and mechanical ball milling. The synergistic effect of the choice of biomass feedstock type and the Fe-Mn loading process together constitute the key variables regulating the physicochemical properties of modified biochar. Variations in the chemical composition, pore structure, and pyrolysis characteristics of different biomass sources influence key properties such as the specific surface area and elemental distribution. These effects occur through mechanisms including elemental migration, carbon skeleton reconstruction, and metal-carbon interactions during the pyrolysis process, ultimately shaping the structure and functionality of the FM-BC material.

### 2.1. Impregnation Pyrolysis Method

The impregnation pyrolysis method involves the in situ loading of metal oxides onto the biochar surface by impregnating biomass with iron and manganese salt solutions, followed by high-temperature pyrolysis (generally above 600 °C). The method is relatively simple, scalable, and widely used for large-scale production. The decomposition kinetics of Fe-Mn precursors during pyrolysis is closely related to the thermal stability of the biochar carbon skeleton [31,32].

For example, Lin et al. [33] treated the pristine biochar by adding it to a mixture of KMnO_4_ and Fe(NO_3_)_3_ solutions for 48 h, and then pyrolyzed it for 1 h at 620 °C under a N_2_ atmosphere for the preparation of FM-BC, and it was found that the specific surface area of FM-BC obtained by the modification reached 208.6 m^2^/g, which was much higher than that of the pristine biochar (60.9 m^2^/g) and that of the Fe-Mn binary oxides (96 m^2^/g). Additionally, the carbon and hydrogen contents decreased, while the nitrogen content increased, contributing to a higher density of active sites for heavy metal adsorption. Zhou et al. [34] prepared the modified FM-BC by impregnating the pristine biochar with the Fe-Mn salt solution at the same mole ratio as Lin et al. and dispersing it with ultrasound for 2 h and then drying it with water for 22 h. FM-BC was prepared by pyrolysis at 0.5 h. It was found that the obtained FM-BC had significantly lower carbon content, higher ash content (14.2 wt%), and higher surface oxygen (35.2 wt%), iron (1.16 wt%), and manganese (7.37 wt%) content compared to pristine biochar, which implies that the FM-BC contains more oxygen-containing functional groups and is more polar. Liang et al. [35] used rice straw as the biomass and loaded FeSO_4_·7H_2_O and MnCl_2_·4H_2_O at different molar ratios to prepare modified biochar. The specific surface area of the modified biochar was significantly enhanced at a pyrolysis temperature of 500 °C, such as FM-BC (an Fe/Mn molar ratio of 3:1) up to 148.155 m^2^/g, which is 17.2 times higher than that of pristine biochar, and the point of zero charge (pHpzc) was increased from 2.29 to 2.58~2.76; metal loading to form Fe_3_O_4_, Mn_3_O_4_, and other oxides was confirmed by SEM and EDS. Fe and Mn were uniformly dispersed to improve the pore structure and enhance the adsorption capacity of atrazine, with the maximum adsorption of FM-BC being 4.3 times that of the original biochar. Despite its advantages, the impregnation-pyrolysis method presents some challenges. Metal dispersion may be uneven, which can be improved by ultrasound assistance or by adjusting the pH of the impregnation solution (typically pH 3–5) [36]. Additionally, the high pyrolysis temperature may damage the biochar’s pore structure, so careful control of the heating rate is necessary to minimize micropore collapse [36].

### 2.2. Hydrothermal Synthesis

The hydrothermal synthesis method facilitates the in situ growth of nano-oxides by combining biochar (or its precursor) with iron and manganese salt solutions under hydrothermal conditions in a sealed autoclave. Typically conducted at temperatures below 200 °C, this method allows precise control over the crystalline structure and morphology of Fe-Mn oxides. One of its key advantages is the ability to retain surface functional groups and active sites, which are often compromised during high-temperature pyrolysis.

Mabagala et al. [37] used rice straw as biomass, which was hydrothermally (100 °C reaction for 8 h) loaded with Fe(NO_3_)_3_·9H_2_O and Mn(NO_3_)_2_·4H_2_O (0.15:0.05 mol/L) to obtain FM-BC. Thus, 10–40 nm nanoparticles were formed on the surface of the modified biochar at the pyrolysis temperature of 700 °C, and crystals such as SiO_2_ and FeAsO were detected by XRD, with good dispersion of the metals; the RSB-Fe/Mn reduced As and Cd in pore water by 67.1% and 80.2%, respectively, during the drought period and increased SOC by 33.9% during the inundation period. The improved pore structure and presence of metal oxides contributed to enhanced heavy metal immobilization and organic carbon preservation. Jung et al. [38] dissolved MnCl_2_·6H_2_O and FeCl_3_·6H_2_O in a solution containing the biochar precursor with stirring until complete dissolution, and then heated the mixture in an autoclave at 180 °C for 10 h after adjusting the pH to 10. FM-BC was obtained, and SEM showed that the surface of the FM-BC was irregular with densely growing agglomerated particles, confirming the presence of the Mn, Fe, C, and O elements, while FTIR showed that cubic spinel-type MnFe_2_O_4_ nanoparticles were successfully attached to the biochar surface. The advantage of the hydrothermal method is that the low-temperature condition can retain the functional groups on the surface of biochar while avoiding the loss of active sites due to high-temperature sintering [39]. Despite its advantages, the hydrothermal method has some limitations. The cost of hydrothermal equipment is relatively high, and precise control of the Fe/Mn molar ratio is essential to prevent phase separation. Additionally, residual by-products such as Cl^−^ and SO_4_^2−^ may remain on the FM-BC surface and must be thoroughly washed away to avoid adverse effects on performance.

### 2.3. Co-Precipitation

The co-precipitation method involves mixing iron and manganese salt solutions with biochar under alkaline conditions so that the iron and manganese ions are synchronously precipitated and attached to the surface and pores of the biochar to form FM-BC. This method is capable of loading nano-sized iron-manganese oxides onto the biochar, and at the same time, it can form a heterojunction structure and enhance the efficiency of electron transfer.

Deng et al. [40] successively combined the biochar with FeSO_4_·7H_2_O and KMnO_4_ solutions and adjusted the pH to 10 for precipitation and drying, and the prepared FM-BC formed Fe/Mn oxides. The specific surface area was enlarged by 50.5 times, and the surface was effectively activated. Lian et al. [41] used soluble starch. FeSO_4_·7H_2_O and MnSO_4_ were homogeneously mixed. KMnO_4_ was added, and the pH of the solution was adjusted to alkaline, and the solution was uniformly stirred, precipitated, and dried to obtain Fe-Mn oxides, and then combined with microorganisms to prepare the FM-oxide-microbial-loaded biochar material, which has better acid resistance, mechanical strength, and mass transfer properties, and a large number of surfaces introduced hydroxyl groups, along with a large number of pores and clefts. Zhou et al. [42] used oak sawn wood as a method of adsorption. They prepared FM-BC from oak sawdust by one-step oxidation/reduction-hydrothermal co-precipitation (6 h at 120 °C) loaded with FeCl_3_·6H_2_O and MnSO_4_·H_2_O (1:1 molar ratio). The concentration increased to 322.1 m^2^/g, and XRD confirmed the formation of mixed crystals of Fe_3_O_4_ and MnFe_2_O_4_ with homogeneous metal dispersion and paramagnetic properties (saturation magnetization strength: 21.5 emu/g); its degradation efficiency of hygromycin at pH = 4.0 reached 98.3%, with a pseudo-first-order kinetic constant of 4.88 min^−1^, and improved the pore structure and metal synergy to enhance the catalytic performance. While the co-precipitation method ensures good metal dispersion and the formation of active nanostructures, the alkaline precipitation environment may lead to partial blockage of the mesopores. To address this issue, the precipitation rate can be modulated using surfactants to preserve the pore network and maintain high surface accessibility.

### 2.4. Sol-Gel Method

The sol-gel method refers to the preparation of stabilized sol by complexing Fe^3+^ and Mn^2+^ with certain stoichiometric ratios, impregnation of biochar, and then aging and drying to form a uniform loading layer, followed by anoxic firing to prepare the FM-BC [43]. Han et al. [44] used egg white as the complexing agent, stirred it to a semi-solid state, and then added 4.7 mL of a 50% manganese nitrate solution and 8.013 g of iron nitrate particles. The mixture was mixed and stirred and sonicated for 30 min. A total of 5 g of corn straw powder was added; it was dried at 60 °C for 12 h and fired at 300 °C under oxygen deficiency conditions for 2 h to prepare FM-BC. The material retained a honeycomb porous structure with a saturated magnetization of 33.19 A/m, enabling rapid separation from solutions under an external magnetic field for desorption regeneration. Its optimal adsorption pH for Zn^2+^ and Cu^2+^ was 5 and 6, respectively, with functional groups like -COOH and -OH facilitating metal complexation. It should be noted that the complexing agent residues may block the pores and need to be thermally activated to remove the organic components; at the same time, sol stability is significantly affected by pH, and the system’s pH needs to be maintained at 4–6 to prevent premature precipitation of metal hydroxides.

### 2.5. Mechanical Ball Milling

Mechanical ball milling is a green and efficient modification strategy to improve the structure of biochar without the need of chemical reagents, and it is often used in combination with other preparation methods. Che et al. [45] showed that after 6 h of ball milling, biochar was successfully loaded with iron and manganese oxides, with the specific surface area 8.11–25.1 times higher (up to 331.5 m^2^/g) than that of pristine biochar due to mechanical force exposing closed pores and alleviating metal-induced pore blockage. It significantly improved the mesoporous structure, with a saturated magnetization of 21.1 emu/g, enabling magnetic recovery for reuse. Ball milling, as an economical and environmentally friendly method, is commonly used in the synthesis of carbon/metal-oxide-based nanoscale advanced materials [46], but the particle size of the raw material needs to be strictly controlled in order to reduce agglomeration, and the mechanical force may destroy the graphitized structure of the biochar, and the optimization of the ball milling time and the filler rate is needed to balance performance and structural integrity.

The physicochemical properties of biochar with different Fe-Mn loading methods are shown in Table 1.

## 3. Removal of Heavy Metal Pollutants by Fe-Mn-Modified Biochar

**Figure 1:**
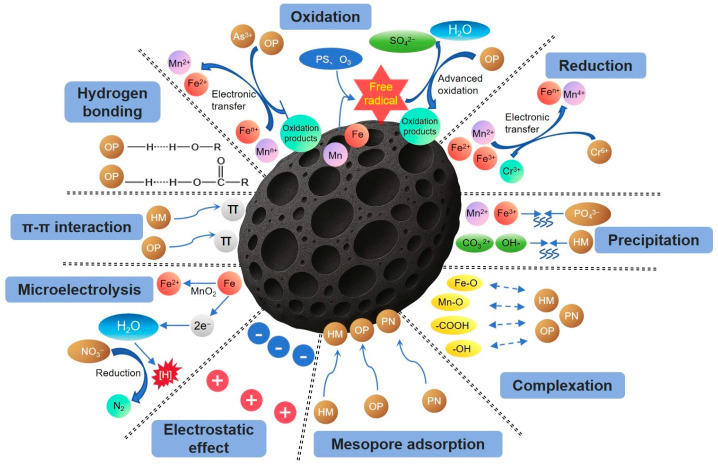
Removal mechanism of heavy metal, organic, inorganic non-metallic salt pollutants by FM-BC.

In the FM-BC system, biochar, as a porous carbonaceous material, has a well-developed pore structure system and a high specific surface area, which provides structured sites for the physical adsorption of heavy metal ions, while Fe-Mn oxides, with their variable valence properties and strong redox capacity, provide diverse chemical mechanisms for the composite material. Based on the porous physical structure of biochar and the redox properties and surface chemistry of Fe-Mn oxides, the composite system can realize the efficient removal of heavy metal ions through multiple mechanisms, such as mesoporous adsorption, redox, complexation, electrostatic force, precipitation, and cation-π (Figure 1), it can effectively remove heavy metals such as As, Cd, Cr, Cu, Hg, Pb, Tl, Zn, etc., from contaminated soils/water bodies (Table 2). Among them, the interactions between different heavy metals and FM-BC have their own characteristics: For Cr(VI), the removal is mainly based on reduction. Low-valent iron, manganese, and nitrogen-containing/oxygen-containing functional groups in FM-BC act as electron donors, reducing Cr(VI) to low-toxic Cr(III) through electron transfer. As(III) tends to be oxidized to As(V) by MnOx in FM-BC. Cations such as Cd^2+^ and Pb^2+^ are prone to complexation and co-precipitation with iron-manganese oxides and oxygen-containing functional groups on the surface of FM-BC, forming Cd_2_Mn_3_O_8_, hydroxides, etc. The adsorption of Hg^2+^ benefits from the optimized mesoporous structure of FM-BC, and the increase in average pore size promotes its adsorption and retention in the pores.

### 3.1. Mesopore Adsorption

Biochar has rich microporous and mesoporous structures, and the Fe-Mn modification method will further optimize the pore distribution, increase the specific surface area, provide more physical adsorption sites, and play a complementary and synergistic role in the removal of heavy metal pollutants. Sun et al. [65] studied the adsorption of Hg^2+^ and Cd^2+^ on FM-BC and found that the specific surface area of the modified biochar decreased from 6.17 m^2^/g to 4.65 m^2^/g, but the average pore size increased from 23.07 nm to 37.03 nm. And the changes in the mesopore structure of Fe-Mn-modified biochar promoted the adsorption of Hg^2+^ and Cd^2+^. Wang et al. [56] investigated the removal of Pb^2+^ and Cd^2+^ by FM-BC and found that the specific surface area of the modified biochar was higher than that of the modified biochar. It was found that the specific surface area of the prepared biochar increased significantly (from 6.53 m^2^/g to 192.41 m^2^/g); the total pore volume (from 0.0116 cm^3^/g to 0.0955 cm^3^/g) and the mesopore structure provided more adsorption sites and shortened the diffusion path of pollutants compared to that before the modification, and the adsorption efficiencies of Pb^2+^ and Cd^2+^ were significantly enhanced. Ferromanganese modification was able to optimize the mesopore structure and change the specific surface area of the biochar, which assisted the synergistic adsorption process. Although the effects varied in different studies, it was effective in shortening the diffusion path of pollutants. In addition, Fe-Mn modification may also affect the diffusion rate and residence time of heavy metal ions in the pores by changing the microenvironment within the pores, which further affects the adsorption effect.

### 3.2. Reduction

Certain heavy metals become significantly more toxic and mobile in higher oxidation states—for example, hexavalent chromium [Cr(VI)] is far more toxic and mobile than trivalent chromium [Cr(III)]. Fe-Mn-modified biochar (FM-BC) offers an effective strategy for detoxifying such species through a synergistic mechanism of reduction and adsorption. Xu et al. [68] found that the zero-valent iron clusters in the FM-BC will form a unique structure during pyrolysis and will reduce Cr(VI) to Cr(III) by electron transfer under the synergistic effect of Mn. The surface Fe-Mn oxides effectively inhibited the passivation of ZVI, and the electron utilization efficiency of Fe for Cr(VI) reduction was enhanced up to 0.08 to 0.19. Chen et al. [62] prepared FM-BC in which Fe(II)-, Mn(II)-, and N-containing nitrogen functional groups acted as electron donors, and Cr(VI) was reduced to Cr(III) by electron transfer under acidic conditions (reduction to Cr(III)), and this reduction process accounted for 72.25% of the removal mechanism of Cr(VI) by FM-BC. Li et al. [63] prepared FM-BC in which Fe(II)-, Mn(II)-, and C=O-containing and other reducing functional groups acted as electron donors and reduced Cr(VI) to Cr(III) under acidic conditions, and the XPS analysis showed a significant reduction effect. More than half of Cr(VI) was converted to Cr(III) in the adsorption process. In summary, FM-BC contains a variety of reducing components, which can reduce the high-valence and highly hazardous heavy metals to low-toxicity forms through electron transfer under the synergistic and acidic environments of low-valent ferromanganese and manganese. This is an effective and unique removal mechanism for high-value and highly toxic heavy metals such as Cr(VI).

### 3.3. Oxidation

In contrast to heavy metals such as chromium, some low-valent metals in the environment are more toxic and more migratory; e.g., the metalloids arsenic and antimony are mostly present in the environment in trivalent and pentavalent forms, and their toxicity strength is inversely proportional to the valence state. FM-BC can be selectively and efficiently removed by the synergistic mechanism of oxidation and adsorption. Xie et al. [69] prepared FM-BC via a microwave-assisted low-temperature oxidation method, which exhibits efficient removal capacity for As(III) in water. MnOx can effectively oxidize As(III) to As(V). The maximum adsorption capacity of this material for As(III) reaches 3.46 mg/g, and there is a synergistic effect in the dual-adsorbate system. Specifically, As(III) can increase the adsorption capacity of Cd(II) by 4.66%, while Cd(II) can increase the adsorption capacity of As(III) by 26.17%. Han et al. [70] found that Mn in FM-BC oxidized As(III) to As(V), while Mn(IV) was reduced to Mn(II) and Mn(IV), creating new adsorption sites for Mn oxides on the surface of Mn(III) and Mn(IV) and further enhancing the adsorption and sequestration capacity. In addition, Sb(III) was also oxidized to Sb(V), and Sb formed Sb-O complexes, which could co-precipitate with Fe(III) minerals. Verma et al. [60] found that in the presence of Mn(IV), As(III) undergoes oxidation, and the reduction of Mn(IV) can precipitate as MnHAsO_4_·H_2_O. Meanwhile, surface complexation with the Mn-OH group on MnO_2_ further eliminates the produced As(V). Collectively, these studies indicate that Mn in FM-BC plays a central role in oxidizing low-valent, highly toxic metalloids such as As(III) and Sb(III). This oxidation not only reduces their toxicity and mobility but also leads to the formation of new active sites for adsorption. The resulting oxidized species can then be effectively removed via co-precipitation, surface complexation, and other physicochemical mechanisms, making FM-BC a multifunctional material for metalloid remediation.

### 3.4. Complexation

The Fe-Mn oxides loaded on the surface of FM-BC and the inherent oxygen-containing functional groups of the biochar can form stable complexes with heavy metal ions through complexation, which can significantly enhance the immobilization capacity of heavy metal pollutants. Wang et al. [71] found that the modified biochar surface showed characteristic peaks of Fe-O (580 cm^−1^) and Mn-O (662 cm^−1^), indicating that Fe^3+^ and Mn^2+^ were loaded on the biochar in the form of oxides and suggesting that these metal oxides form complexes with the oxygenated anions of Cr(VI) through ligand exchange reactions. The loading of Fe-Mn oxides also promotes the formation of the mineral phase and further enhances the complexation process. Chu et al. [72] studied the removal of Cr(VI) using FM-BC; it was found that Fe_2_O_3_, MnCO_3_, and Fe-Mn complex oxides in FM-BC could undergo ligand exchange with reduced Cr(III) during adsorption via Fe/Mn-O-Cr bonding to produce an ≡Fe/Mn-O-Cr^3+^ ternary complex. Similarly, Yang et al. [73] observed crystalline phases of CdCO_3_ and Cd_2_Mn_3_O_8_ in FM-BC after Cd adsorption, suggesting that Cd^2+^ undergoes a dual role of co-precipitation and complexation with CO_3_^2−^ and Mn-O structures. In summary, the Fe-Mn oxides on FM-BC surfaces, combined with the oxygen-containing functional groups of biochar, generate stable complexes with heavy metal ions primarily via ligand exchange and co-precipitation, thereby greatly enhancing the material’s heavy metal immobilization capacity.

### 3.5. Electrostatic Effect

FM-BC plays an electrostatic role in the heavy metal removal process through the modulation of surface charge properties, which mainly involves the electrostatic attraction and repulsion effects between surface functional groups and heavy metal ions. Liang et al. [74] found that the adsorption mechanism of Fe-Mn dibasic oxides/manganese biocarbon composite adsorbents for Cr(VI) mainly includes electrostatic interactions and so on. When the solution’s pH was lower than the zero-charge point of FM-BC (pH_zpc_ = 7.4), its surface was positively charged, and Cr(VI) in the solution mainly existed in the form of anions, such as HCrO_4_^−^ and Cr_2_O_7_^2−^, which were adsorbed onto the surface of FM-BC by electrostatic attraction, thus realizing the removal of Cr(VI). Sun et al. [65] prepared FM-BC for adsorption of Cd^2+^, and it was found that the same significant electrostatic effect existed during the adsorption process, and the initial solution’s pH changed the surface charge of FM-BC, and under alkaline conditions, the positive charge on the surface of FM-BC was reduced, which was favorable for the adsorption of the positively charged Cd^2+^, and the removal of Cd^2+^ reached a peak when the pH exceeded 7, highlighting pH-dependent electrostatic modulation. Moreover, electrostatic action may have a synergistic effect with other adsorption mechanisms, such as combining with mesoporous adsorption, so that heavy metal ions with a specific charge are guided by electrostatic gravity and directed more efficiently into the pore space of the biochar, and because of the pore surface charge characteristics and the fit of the ionic charge, the ions are allowed to fit more tightly onto the surface of the pore space, which significantly enhances the strength of the adsorption of heavy metal ions on the surface of the pore space [48].

### 3.6. Precipitation

The removal of heavy metals such as Cd and Pb mainly depends on the co-precipitation of iron and manganese oxides on the surface of the biochar. Qu et al. [58] showed that FM-BC remove Cd^2+^ from water through mechanisms such as chemical precipitation. Their adsorption capacity is 4.8–6.1 times that of pristine biochars. For example, the adsorption capacity of Fe/Mn-BMRH for Cd^2+^ can reach 53.92 mg/g, and the adsorption capacity increases with the rise in pH value, which confirms the significant effect of precipitation on pollutant removal. Yang et al. [50] found that after the adsorption of Cd onto the FM-BC, the diffraction peaks of CdCO_3_, Cd_2_Mn_3_O_8_, and CdO were detected, indicating that Cd co-precipitates with the mineral constituents in the FM-BC during the adsorption process. Wang et al. [56] found by EDS analysis that the elemental peaks of K and Ca on the surface of the material disappeared after the adsorption of Pb(II) on MF-S@CBC and MF-OH@CBC, and that when the solution was in an alkaline environment, Pb(II) could easily bind with hydroxide ions and then form a precipitate. Chen et al. [62] found that under acidic conditions, the Fe(II)-, Mn(II)-, and FM-BC-containing N functional groups acted as electron donors to reduce Cr(VI) to Cr(III), which was readily hydrolyzed to produce Cr(OH)_3_ and Fe(III)/Cr(III) hydroxides with increasing pH, resulting in co-precipitation for further chromium removal. Precipitation can effectively reduce the mobility and bioavailability of heavy metals in the environment and promote the solidification and stabilization of heavy metals from the level of chemical-form transformation and physical immobilization.

### 3.7. Cation-π Action

The cation-π effect mainly originates from the aromatic carbon structure of biochar, whose π-electron system can be electrostatically attracted or orbitally hybridized with heavy metal cations, thus enhancing the adsorption capacity. High-temperature pyrolysis can significantly enhance the degree of aromatization of biochar and form polycyclic aromatic hydrocarbon structures. Tang et al. [66] found that the adsorption capacity of FM-BC for Pb^2+^ was enhanced by about 2.36-fold compared with that of pristine biochar through Langmuir model fitting, and the intensity of -OH, O=C-O, and C=C peaks of adsorbed alcohols and phenols was decreased, suggesting that chemical bonding was involved in the adsorption process, whereas the involvement of C=C bonding reflects that the synergistic effect of Fe/Mn oxides and aromatic carbon may have played a contributing role to some extent in the enhancement of cation-π bonding. In addition, FM-BC would convert heavy metal ions into Fe-Mn-oxide-bound states in soil through charge transfer of the π system of aromatic carbon with metal ions.

## 4. Removal of Organic Pollutants by Fe-Mn-Modified Biochar

Biochar can effectively remove organic pollutants. For instance, the unmodified biochar prepared from corn cobs under the pyrolysis condition of 900 °C for 2 h can achieve good adsorption capacity for delafloxacin [3]. The Fe-Mn oxides loaded on FM-BC can significantly enhance the chemical degradation of organic pollutants by virtue of their own oxidizing properties and their activation of strong oxidants such as persulfate—this advanced oxidation driven by Fe-Mn synergism constitutes a unique interaction mechanism for organic substances. In addition to oxidation, the removal of organic pollutants by FM-BC also includes mechanisms such as mesoporous adsorption, complexation, hydrogen bonding, electrostatic, and π-π EDA mechanisms of action (Figure 1), and the removal effect is also significant (Table 3).

### 4.1. Mesoporous Adsorption

Mesoporous adsorption plays a significant role in the degradation of organic pollutants. On the one hand, the mesoporous structure of biochar provides attachment sites for Fe/Mn ions, which reduces the generation of the passivation layer on the surface of the material and increases the contact area of active radicals with organic pollutants; on the other hand, Fe/Mn ions generated from the corrosion of Fe-Mn bimetallic particles during the preparation process are adsorbed onto the surface of biochar, which results in the formation of a large number of micropore pores on the surface of the biochar, which further facilitates the degradation of the organic pollutants [78]. Chang et al. [84] observed by scanning electron microscopy that the surface and pores of BC and FM-BC adhered to soil mineral particles after remediation, and these pore changes affected their adsorption of dibutyl phthalate (DBP) and bis (2-ethylhexyl) phthalate (DEHP) in soil, resulting in an increase in the residual amount of DBP and DEHP in the soils treated with BC and FM-BC, which suggests that the mesoporous structure provides an opportunity for pollutant attachment and adsorption. The structure provides space for the attachment and storage of pollutants and plays an important role in the adsorption and immobilization of pollutants. Ma et al. [81] investigated the mesoporous adsorption mechanism of FM-BC. N_2_ adsorption/desorption and pore-size distribution curves of SBC and Fe/Mn-SBC were analyzed, and the results showed that the N_2_ adsorption/desorption isotherms of both belonged to the typical type IV isotherms with H3 hysteresis loops, which indicate that the mesopore is the main pore structure. It provides more active sites for the reaction and can realize the efficient degradation of sulfamethoxazole. Although mesopore adsorption is usually not the main pollutant removal method, the mesopore structure promotes the contact between organic pollutants and active sites to a certain extent and plays a synergistic and positive effect on the overall degradation reaction.

### 4.2. Oxidation

FM-BC removes organic matter by activating free-radical- or non-free-radical-mediated oxidation reactions, where the advanced oxidation reaction generated by free radicals is a unique mechanism for the degradation of organic pollutants. Jiao et al. [95] prepared industrial lignin-based FM-BC, which can efficiently degrade oxytetracycline (OTC) by activating persulfate. The oxygen-containing functional groups in industrial lignin, the synergistic effect of Fe and Mn, and defective structures can serve as active sites to generate reactive radicals such as SO_4_^−^· and ·OH. This material can achieve a 90% degradation rate of OTC within 30 min and has good stability. Li et al. [90] found in their study on the degradation of tetracycline that the Fe/Mn-OH site acts as a key reactive center to activate peroxynitrite (PS) through electron transfer, which leads to the decomposition of PS to generate sulfate radicals (SO_4_^−^·), hydroxyl radicals (·OH), and singlet oxygen (^1^O_2_), and these reactive oxygen species attacked the conjugated double bonds, amino groups, and other functional groups in the tetracycline molecule through oxidation, leading to the fracture of its molecular structure and its eventual mineralization into CO_2_ and H_2_O. Xiao et al. [89] found that, in the degradation of bisphenol A (BPA), Fe/Mn binary metal synergism accelerated the O-O bond breaking of PS and promoted the generation of SO_4_^−^· and ·OH with a degradation rate constant as high as 1.7337 min^−1^, which was 20 and 91 times higher than that of single-metal Fe or Mn catalysts, respectively, confirming that the synergistic effect of bimetallic oxidation significantly enhances the efficiency of radical generation. FM-BC can also activate ozone to produce advanced oxidation reactions. Xu et al. [75] found that under the conditions of an FM-BC concentration of 0.5 g/L, a pH value of 7, and an ozone dosage of 4.93 mg/min, more than 95% of 50 mg/L ibuprofen and 80.5% of the total organic carbon can be removed within 9 min. The reaction rate is 6.58 times that of ozone oxidation alone and 2.3–4.1 times that of other catalysts. It degrades pollutants mainly through superoxide radicals and hydroxyl radicals. In the degradation of reactive blue 19 (RB19), Qiu et al. [83] further found that upon activation of persulfate by Fe/Mn-BC, in addition to the radical pathway, high-valent Fe(IV)/Mn(VII) may directly attack the conjugated structure of the dye molecule through non-radical oxidation, forming coordination complexes and triggering electron transfer, thus synergistically enhancing the degradation effect. These studies suggest that oxidation involves both advanced oxidation reactions based on activation-mediated generation of free radicals such as persulfate and direct electron transfer oxidation of high-valent metal ions, and that the two roles together drive efficient degradation of organic pollutants.

### 4.3. Complexation

Complexation is mainly reflected in the formation of coordination bonds between metal sites on the surface of FM-BC and functional groups of organic pollutant molecules, which enhances the adsorption and degradation efficiency. Alazba et al. [93] observed in the adsorption study of methylene blue (MB) by FTIR spectroscopy that the intensity of Fe-O and Mn-O vibration peaks was significantly reduced after adsorption, which indicated that the nitrogen and oxygen functional groups of MB molecules were associated with Fe^3+^ and Mn^2+^ to form surface complexes. This coordination resulted in the 100% removal of MB by FM-BC, which was significantly higher than that of 25% for unmodified biochar. Xiang et al. [94] in levofloxacin adsorption showed that the carbonyl (C=O) and amino (-NH_2_) groups in LEV molecules can act as ligands with the metal oxide active sites on the surface of FM-BC (e.g., Fe^3+^ and Mn^4+^) to form coordination complexes, and this effect was particularly significant under acidic conditions (pH = 5), which prompted a maximum adsorption capacity of 181 mg/g. In addition, Tao et al. [76] found in the adsorption of estrone that metal hydroxyl (M-OH) groups on the surface of Fe/Mn-BC could form coordination bonds with the hydroxyl groups of E1, which enhanced its adsorption capacity in the aqueous phase, especially at low ionic strength, and complexation became one of the dominant mechanisms. Complexation not only enhances the adsorption affinity of pollutants but also promotes the subsequent oxidation reaction through the activation of the metal center, forming a synergistic process of “adsorption-complexation-oxidation”.

### 4.4. Hydrogen Bonding

In the FM-BC system, hydrogen bonding is mainly manifested in the directional binding of oxygen-containing functional groups (e.g., hydroxyl and carboxyl groups) on the surface of the biochar with the polar groups of the pollutants. For example, Huang et al. [80] found that hydrogen bonding between hydroxyl and other groups on the surface of FM-BC, the amide group of sulfamethoxazole, and the specific polar group of polystyrene microplastics, respectively, facilitated the adsorption and removal process of both on FM-BC. Song et al. [96] found that the addition of FM-BC to soil contaminated with chlorinated organic matter can adsorb the pollutants in a targeted manner through hydrogen bonding, which can effectively reduce the length of the active substance transport path, and significantly increase the frequency of collision contact between chlorinated organic pollutants and the active substance in the soil. In addition to the direct role played by hydrogen bonding, in the water environment reaction system, water molecules are widely present. ·OH and other free radicals may form hydrogen bonds with water molecules, affecting their distribution and activity in the system, which in turn affects the degradation efficiency of the organic matter, and there may also be hydrogen bonding between the surface functional groups of the biochar and the free radicals, which stabilizes the free radicals and enhances their oxidative capacity towards the organic matter [86].

### 4.5. Electrostatic Effect

Electrostatic attraction is a key mechanism for the removal of anionic pollutants by FM-BC. Alazba et al. [93] found that at lower pH values, the surface of FM-BC was enriched with H^+^ ions, which were positively charged, and there was an electrostatic repulsion between them and the cationic dye, MB, hindering the adsorption of MB; with the increase in pH, the concentration of H^+^ ions decreased, and the electrostatic repulsion was weakened, which was favorable to the adsorption of MB. Huang et al. [80] found that when they investigated the mechanism of competition for adsorption of FM-BC systems between polystyrene microplastic (PS-MP) and sulfamethoxazole (SMX), they found that PS-MP was negatively charged in a wide pH range, and SMX gradually existed in anionic form when the pH was >5.6, and the negatively charged SMX electrostatically repelled the negatively charged FM-BC when the pH was ≈10, resulting in the lowest removal of SMX, which proved the important influence of the electrostatic effect on the adsorption effect. The electrostatic effect will also play an indirect promotion effect. Xu et al. [75] found that the protonated hydroxyl group on the surface of FM-BC has a positive charge, while the ozone molecule has a certain polarity, and it is part of the charge distribution and is not uniform, and the attraction based on the charge between the two produces the electrostatic effect, so the ozone molecule can be close to and adsorbed in the vicinity of the hydroxyl group on the surface of the catalyst, and this electrostatic effect creates a favorable condition for the subsequent chemical reaction, which makes it easier for ozone molecules to participate in the adsorption process. It also makes it easier for ozone molecules to participate in the free-radical chain reaction, which promotes the generation of reactive oxygen species (ROS), and thus achieves the goal of effective degradation of ibuprofen.

### 4.6. π-π EDA Interaction

π-π interactions play an important role in the adsorption of aromatic pollutants on FM-BC. Huang et al. [80] found from FTIR spectroscopic data that the position and intensity of the peaks related to the aromatic structure of the FM-BC were changed after sulfamethoxazole adsorption; for example, the peak at 1584 cm^−1^ was weakened, confirming that the π-π EDA effect was involved in adsorption and affected the performance of SMX removal. Meanwhile, the off-domain π-electrons in Fe/Mn co-doped biochar could promote the decomposition of ozone into hydroxyl radicals (·OH) and superoxide radicals (·O_2_^−^) by enhancing the charge transfer ability of the carbon skeleton, thus significantly enhancing the degradation of organic pollutants such as ibuprofen in the catalytic ozonation process [75]. Chang et al. [84] found that the stretching vibrations of the FM-BC aromatic skeleton (C-C and C-O) at about 1560 cm^−1^ turned blue after repair, whereas the stretching vibrations were associated with the alkanes C-C, C-O-C, and Si-O. The absorption peak at 1050 cm^−1^ shifted to a lower wave number, suggesting a possible relationship with π-π interactions that are possibly involved in the ligand adsorption process.

## 5. Removal of Inorganic Non-Metallic Salt Pollutants by Fe-Mn-Modified Biochar

At present, there are few research results on FM-BC in the field of inorganic non-metallic salt pollutant degradation in the world, and at this stage of research, it is shown that FM-BC is also effective in removing phosphate and nitrate pollutants (Table 4), and the removal mechanism is similar to that of heavy metals and organics, which includes mesoporous adsorption, electrostatic, precipitation, complexation, and microelectrolytic effects (Figure 1).

### 5.1. Mesoporous Adsorption

Suitable mesoporous structures include FM-BC prepared under the condition of the FeCl/KMnO_4_ solution with a molar ratio of 1:1; the average pore diameter will be larger than the ionic radius of nitrate, which helps nitrate enter the interior of the pore, increases the contact area with the adsorbent, and promotes the adsorption effect, and at the same time, the mesoporous structure also works synergistically with the other adsorption mechanisms and jointly improves the effect of adsorption on the salt substances [101]. It has also been shown that the specific surface area of iron-manganese-modified ball-milled biochar can reach 226.5–331.5 m^2^/g, which is 8.27–15.1 times higher than that of the original biochar, and its adsorption capacity of phosphate is 4.47–5.82 times higher than that of the original biochar [45]. Fu et al. [97] found that iron-manganese synergistic modification effectively increased the specific surface area and porosity of the microalgae biochar, and its specific surface area and pore volume increased four times compared with the traditional microalgae biochar, which enhanced its adsorption capacity for phosphate, and the material had a mesoporous structure with a pore size in the range of 2–50 nm.

### 5.2. Electrostatic Effect

Under the influence of iron-manganese modification, the surface properties of biochar are changed so that it is positively charged under specific conditions and electrostatically attracted to negatively charged phosphate or nitrate ions. Zheng et al. [101] found that when the solution’s pH is less than the zero-charge point (pHpzc) of the FM-BC composite, the surface of the adsorbent is positively charged and is able to adsorb nitrate anions in solution by electrostatic attraction. Che et al. [45] found that the surface of the FM-BC is protonated and positively charged at low pH, which is conducive to the adsorption of negatively charged phosphate anions, and as the pH increases, the surface negative charge increases, the electrostatic repulsion of phosphate is enhanced, and the adsorption decreases. Fu et al. [97] prepared FM-BC with a zero-charge point of 4.12; when the solution’s pH is lower than pHpzc, the surface of FM-BC is protonated and positively charged, and the phosphate ion is negatively charged, and an electrostatic attraction between the two will be generated, which promotes the adsorption of phosphate. When the solution’s pH = 2~3, the surface of FM-BC is positively charged, which has a strong electrostatic adsorption effect on the anionic form of phosphate; when the solution’s pH is higher than pHpzc, the surface of FM-BC is negatively charged, which produces electrostatic repulsion with phosphate ions, which is unfavorable for the adsorption process, and the concentration of hydroxide ions increases at this time, which competes for the adsorption site with phosphate ions and further reduces the adsorption efficiency.

### 5.3. Precipitation

Iron and manganese ions on the surface of FM-BC will react with phosphate ions under certain conditions to produce insoluble phosphate precipitates. When the concentration of phosphate ions in the solution is high, ions such as Fe^3+^ and Mn^2+^ may combine with phosphate to form precipitates such as FePO_4_ and Mn_3_(PO_4_)_2_, thus reducing the concentration of phosphate in the solution and realizing the removal of phosphate. This process was somewhat verified in the SEM observation, where a large number of irregular and rough particles appeared on the surface of FM-BC after adsorption, suggesting possible precipitation generation [45,97]. Beiyuan et al. [98] showed that the removal of phosphate by FM-BC (containing Fe_3_O_4_ and MnO_2_) mainly relies on mechanisms such as precipitation, with a maximum adsorption capacity of 17.93 mg/g. In contrast, FM-BC(containing MnFe_2_O_4_) has a smaller contribution from precipitation, but its maximum adsorption capacity reaches 135.88 mg/g. Additionally, the adsorption capacity of Fe/MnBC drops sharply at pH = 12, confirming the significant impact of precipitation on pollutant removal under specific conditions. At the same time, the Fe-O and Mn-O bonds on the surface of FM-BC and phosphate will undergo a coordination reaction to form endo-sphere complexes (e.g., Fe-O-P and Mn-O-P bonds), and the hydroxide colloid generated from the hydrolysis of the metal ions under neutral to alkaline conditions can capture the phosphate by adsorption or co-precipitation, thus exerting the role of precipitation in removing the phosphate pollutants [102].

### 5.4. Complexation

There are abundant functional groups on the surface of FM-BC; when adsorbing phosphate, H_2_PO_4_^−^ and -OH exchange ligands to form inner-sphere complexes, prompting a rise in the solution’s pH, which improves adsorption capacity and alters the solution’s pH [45]. In the case of nitrate adsorption, complexation is also significant. Depending on the relationship between the solution’s pH and the zero-charge point, nitrate undergoes ligand exchange with OH- on the surface to varying degrees, which leads to the formation of stable complexes with Fe-Mn oxides for adsorption [101]. The researchers provided strong evidence for the occurrence of complexation by FTIR and XPS analyses. FTIR analyses revealed that the hydroxyl peak of FM-BC (3367 cm^−1^) was weakened and shifted after the adsorption of phosphate, and a new P-O bond bending vibrational absorption peak (1052 cm^−1^) appeared, suggesting that complexation with the functional group had occurred. XPS analyses also confirmed that metal oxides, hydroxyl groups, and the oxidized surface OH- were exchanged to different degrees. This confirmed the involvement of metal oxides, hydroxyl groups, etc., in the adsorption process, suggesting the formation of complexes between phosphate and these groups and resulting in the adsorption of phosphate [97].

### 5.5. Microelectrolysis

Inorganic non-metallic salts exist in the form of stable ions and need to be removed through ion-form transformation, while microelectrolysis can generate reductive substances and metal ions through spontaneous redox reactions, directly realizing their reductive transformation and precipitation separation. The precise matching between this mechanism and the characteristics of inorganic salts makes it applicable [103]. Microelectrolysis exists in specially constructed FM-BC systems, such as in the sponge iron/biochar/manganese sand system, which will spontaneously form a tiny primary cell and generate microelectrolysis. Sponge iron acts as the anode, and Fe causes the loss of electrons to be oxidized; biochar acts as the cathode, and electron transfer and the water reaction are needed to generate strong reducing [H]. [H] can promote nitrate reduction, improve nitrogen selectivity, and play the role of the significant enhancement of the nitrate removal rate; when the ratio of sponge iron and biochar is 3:1, the removal rate is 61.5%, which is far more than the sum of the two alone, and the maximum selectivity of nitrogen is up to 22.4%, while the pure sponge iron system is only 5.8%. The addition of manganese sand will further strengthen the microelectrolysis process, in which MnO_2_ accelerates the conversion of Fe^2+^ into Fe^3+^, reduces the passivation of sponge iron, and accelerates electron transfer [100].

## 6. Other Effects of Fe-Mn-Modified Biochar on the Environment

Biochar can improve soil permeability, regulate trace elements, promote nutrient cycling, and effectively increase the organic matter content, nitrogen fixation, and the nitrification rate [104,105], while FM-BC, because of the physicochemical properties of biochar, will have the same positive impact on the soil environment, providing a good growing environment for plants and promoting the development of plant roots and nutrient uptake. In terms of soil physicochemical properties, FM-BC enhances soil pH, reduces redox potential (Eh), and promotes the stabilization of some pollutants by releasing alkaline components and optimizing the pore structure. For example, Gao et al. [106] found that pH increased by 0.42 ± 0.07–1.15 ± 0.16 and 0.38–1.15 ± 0.05 after the application of FM-BC to dibutyl phthalate (DBP)- and di(2 ethylhexyl) phthalate (DEHP)-contaminated soils, respectively. Sun et al. [107] demonstrated that the decrease in soil Eh was greater in FM-BC treatments than in the original biochar. This is due to the extensive pore structure and specific surface area of FM-BC, which are conducive to the improvement of soil aeration and the water-holding capacity, and the introduction of unstable organic compounds into FM-BC enhanced microbial respiration and accelerated the consumption of soil O_2_, which reduced soil Eh; at the same time, FM-BC enriched nutrients such as organic carbon (SOC), effective nitrogen (AN), phosphorus (OP), and potassium (AK), and the Hg/Cd-contaminated soil SOC content increased by 24.61–44.21%, and effective potassium increased by 212%, providing the material basis for the soil ecosystem. At the biochemical level, FM-BC can activate the activities of oxidoreductase enzymes such as catalase (CAT) and polyphenol oxidase to enhance the oxidative capacity of the soil and promote the activities of enzymes involved in nitrogen and phosphorus cycling such as urease and neutral phosphatase to optimize the efficiency of nutrient conversion. In terms of the microbial community structure, FM-BC can enrich Proteobacteria, Firmicutes and other advantageous flora; reduce Bacteroidetes; and promote the proliferation of Pseudomonas and other functional bacteria to strengthen the metabolic pathway related to pollutant degradation and nutrient cycling [108]. In terms of the plant growth response, FM-BC promotes the biomass accumulation of rice and wheat by improving the inter-root microenvironment; for example, it can increase the dry weight of rice roots and grains by 14.6–36.7% and induce the formation of iron-manganese plaques in the root system, which can effectively prevent the migration of heavy metals to the surface [107]. It can be seen that FM-BC can effectively improve the soil environment and promote plant growth while degrading pollutants, which has important agricultural application value.

## 7. Prospects for Recycling of Iron-Manganese-Modified Biochar

Regeneration of biochar material is conducted to restore the composite material that has reached saturation adsorption to its original state through physical and chemical methods [109]. Iron-manganese-modified biochar can inhibit the structural collapse of the biochar skeleton during regeneration due to the presence of ferromanganese oxides, thus maintaining the stability of its porosity and surface active sites, while the redox properties of ferromanganese can restore the adsorption sites through electron transfer, further extending the material’s lifetime. Li et al. [110] found that the reduction of Mn to Mn(III) and Mn(II) exposed new adsorption sites of the biochar, and the pore structure was intact and retained, and pore filling and electrostatic attraction were able to continue to play a role in facilitating the continuation of the reaction when stabilizing arsenic in the soil by using the FM-BC solidification method. The recycling of iron-manganese-modified biochar is promising, and it can realize the closed-loop goal of “function reuse-resource regeneration-environmental gain” in the fields of water-soil-energy through the paths of ultrapure water purification, acid and alkali treatment, and magnetic power separation.

### 7.1. Ultrapure Water Purification

Ultrapure water purification is a common regeneration method, and FM-BC can be effectively rinsed out of various impurities and pollutants adsorbed in the pore structure with deionized water so that the adsorption sites of FM-BC can be re-exposed to restore its adsorption capacity. Qu et al. [58] rinsed and recycled FM-BC with deionized water, and after five cycles of adsorption and desorption, the FM-BC still retained 41–70% of its capacity compared to the first adsorption, but this adsorption capacity is still very low. The adsorption capacity of FM-BC was still larger than that of the original biochar. Chen et al. [92] synthesized FM-BC with the Fe and Mn elements in various valence states, and oxygen-containing functional groups on the surface were recovered by rinsing with deionized water, and it still showed high catalytic activity after five times of reuse and less leaching of metal ions, which made it a very promising catalyst for peroxodisulfate activation. The purification of ultrapure water is simple, and the reagent’s requirement is not high, but at the same time, it will lead to serious loss of effective biochar particles, and the agglomeration phenomenon will bring the problem of low recycling efficiency, as well as larger water consumption and a higher time cost.

### 7.2. Acid and Alkali Treatment

Acid-base treatment can effectively regenerate FM-BC through the ion-exchange reaction between H^+^ in acid and adsorbed heavy metal ions, as well as the neutralization reaction between OH^−^ in alkali and acidic pollutants, and the repair of the blocked pore structure and the adsorption rate can be maintained at more than 60% after saturated regeneration of adsorption of heavy metals in five cycles. Verma et al. [60] used a 0.1 M NaOH solution as the eluent for five cyclic regenerations of FM-BC. The adsorption capacity decreased to 78% of the initial value but still maintained good cyclic stability. The capacity loss was mainly attributed to the partial dissolution of iron-manganese oxides and the consumption of surface active sites. Zhu et al. [64] used the 0.5 mol/L NaOH desorption method for FM-BC. The FM-BC was regenerated using 0.5 mol/L NaOH desorption, and its removal efficiency of Cr(VI) could still reach 60.76% in the sixth cycle, indicating that the prepared FM-BC still has certain reuse value, and NaOH can effectively regenerate it. The composite adsorbent of activated carbon/ferromanganese oxide prepared from mulberry sticks by Qin et al. [111] showed good regeneration performance, and the FB-BC was regenerated by 0.5 mol/L NaOH, and after three adsorption-desorption regenerations, the adsorption amount of Cr(VI) was only reduced by 13.2%, and the regeneration and utilization efficiency reached 86.8%. Nitric acid treatment helps to increase the number of acidic groups on the surface of FM-BC, which in turn promotes the adsorption of heavy metals. Tan et al. [61] used a 1 mol/L nitric acid solution to elute BC-FM after adsorption of Cd(II), and the mixture was oscillated in a constant-temperature oscillator for 24 h. The mixture was washed with deionized water to a constant pH, and the regenerated BC-FM was obtained after drying, and after repeating three sets of adsorption cycles, the removal rate of Cd was maintained at 45.4–66.2%. Xiao et al. [51] considered that the leaching of Fe and Mn would destabilize the material. The KH_2_PO_4_ solution with acid-base buffering properties was chosen as the desorbent in the study, and the removal rates of Cd(II), Cu(II), and Pb(II) were still maintained in FM-BC after four cycles. Removal efficiency remained above 70%.

### 7.3. Magnetic Separation

The ferromanganese oxides introduced during the preparation of FM-BC magnetize the biochar to form a relatively stable magnetic structure, which is not easily interfered by external factors and loses its magnetism under the general environment and conditions of use, and using this feature, FM-BC can be recycled and used by magnetic separation. Zhao et al. [47] successfully synthesized a new type of KMnO_4_-modified lucerne biochar with loaded nano-Fe_2_O_3_(FMLB)-modified lucerne biochar with a saturation magnetization strength of 10.41 emu/g, which was easy to separate from the aqueous solution using a magnet, and its adsorption performance for Cu(II) remained above 75% after four adsorption cycles. Ma et al. [81] found that the surface of the prepared Fe/Mn bimetallic co-functionalized sludge biochar had a characteristic diffraction peak of magnetism, with a saturation magnetization strength value of 9.31 emu/g, and the degradation rate of sulfamethoxazole by the EO/Fe/Mn-SBC/Na_2_SO_4_ system with it as the activator still reached up to 79.5% after five times of reuse. Yu et al. [55] used a magnet to separate the FM-BC in an aqueous solution, and then regenerated the adsorbed FM-BC by shock desorption; although the removal efficiency of FM-BC for Cr(VI) decreased with the increase in the number of regeneration times in the fifth adsorption experiment, the removal rate still reached 82.34%; when the Cr(VI) content was reduced to 5 mg/L, the removal rate of Cr(VI) by FM-BC after five adsorption cycles was as high as 97.68%.

### 7.4. Comparative Analysis of Regeneration Methods

The three regeneration methods above for FM-BC each have their own focuses: Ultrapure water purification is simple to operate but water- and time-consuming, with a tendency for particle loss, limiting its large-scale application. Acid and alkali treatment exhibits relatively good cyclic performance, yet it has issues like metal leaching and waste liquid disposal, requiring cost control for medium-scale applications. Magnetic separation enables efficient recovery by virtue of magnetism, maintaining over 75% efficiency after 4–5 cycles, facilitating automation, and being more suitable for large-scale applications, though it has higher energy consumption during preparation.

Comprehensively, magnetic separation is superior in terms of environmental friendliness and scalability. Acid and alkali treatment, under controlled conditions, is suitable for medium- and high-pollution scenarios, while ultrapure water purification is only appropriate for small-scale temporary use. Meanwhile, research on the long-term performance of FM-BC can be further advanced. Existing studies have shown that FM-BC can still maintain high pollutant removal efficiency (e.g., the removal rate of hexavalent chromium reaches over 78.9%) after long-term interaction with oxidants and natural oxidation (for 1–3 months). Its embedded zero-valent iron clusters, due to their unique structure, possess strong resistance to oxidative passivation, demonstrating excellent long-term stability [68]. Next, it is necessary to optimize reagents and equipment to balance efficiency, environmental friendliness, and durability.

## 8. Conclusions and Outlook

As an emerging environmental functional material, FM-BC has demonstrated considerable potential in heavy metal remediation owing to its unique physicochemical characteristics and multi-mechanism synergistic effects. By incorporating bimetallic iron and manganese into the biochar matrix, FM-BC establishes a redox-active system that leverages the complementary functionalities of both metals. Compared with single-metal iron-modified biochar and the original biochar, FM-BC can effectively overcome the technical bottlenecks such as low passivation efficiency, insufficient structural stability, and a single reactive site. The synergistic effect of iron and manganese significantly improved the adsorption capacity and removal efficiency of the material to a variety of pollutants.

Despite promising laboratory-scale advances, several limitations hinder the broader application of FM-BC. At the fundamental level, the influence of microstructure and surface chemistry on adsorption performance remains insufficiently understood. Key factors such as metal loading morphology, distribution uniformity, and interactions between metal species and surface functional groups profoundly affect the adsorption kinetics and thermodynamics, yet a comprehensive theoretical framework has not been fully established. From a practical standpoint, most current studies are confined to simulated contamination systems. In real-world environments, the high heterogeneity of soil physicochemical properties and the complexity of coexisting pollutants may significantly influence the performance of FM-BC. Therefore, its field effectiveness, stability, and long-term remediation capacity require further validation. Moreover, challenges related to cost control, large-scale production optimization, and the assessment of secondary pollution risks continue to limit the transition of FM-BC from laboratory research to industrial-scale application. Although the potential environmental risks associated with the reuse or disposal of spent FM-BC (e.g., leaching of adsorbed contaminants) appear minimal, explicitly evaluating such scenarios would enhance the robustness and sustainability of its application framework.

Future research on FM-BC can advance along the following four core directions:

1. Microstructural optimization and adsorption enhancement: To deepen understanding of the structure-activity relationship in FM-BC, advanced characterization techniques such as high-resolution transmission electron microscopy (HRTEM), X-ray photoelectron spectroscopy (XPS), and synchrotron radiation should be utilized. These tools can elucidate the links between crystal structure, pore architecture, elemental valence distribution, and adsorption behavior. By systematically regulating key preparation parameters—such as pyrolysis temperature, Fe/Mn molar ratio, and the type of activating agent—researchers can fine-tune the pore structure and surface functional properties of FM-BC. Such targeted modifications can lead to enhanced adsorption capacities and more stable pollutant immobilization, thereby minimizing the risk of secondary pollution due to leaching.
2. Expansion of feedstocks and preparation methods: The feedstock base for FM-BC should be broadened beyond conventional agricultural and forestry residues. Unconventional biomass sources, such as algae, sewage sludge, and industrial organic waste, offer promising alternatives with unique physicochemical properties. Concurrently, the development of low-cost and energy-efficient synthesis methods, including co-pyrolysis with waste-derived additives, can reduce production costs and promote circular resource utilization. Capitalizing on the inherent functional groups and mineral compositions of these novel feedstocks may enable the fabrication of FM-BC with tailored adsorption functionalities, making it better suited for the removal of specific or complex pollutant mixtures.
3. Engineering applications and field validation: To bridge the gap between laboratory findings and real-world deployment, greater emphasis must be placed on pilot-scale studies and field trials in contaminated sites such as mining regions, agricultural lands, and industrial zones. These studies should assess FM-BC’s long-term stability, pollutant retention performance, and ecological safety under variable environmental conditions. A particularly promising direction is the integration of FM-BC into permeable reactive barrier (PRB) systems. Given its porous structure and high pollutant affinity, FM-BC can serve as an effective filler material for continuous in situ groundwater remediation across large areas. Previous studies have demonstrated the feasibility of using metal-modified biochar in PRB systems for sustained contaminant removal, underscoring the engineering potential of FM-BC for site-specific applications.
4. Comprehensive policy and economic considerations: Economic feasibility is pivotal for the large-scale adoption of FM-BC. Cost reductions can be achieved by coupling low-cost raw materials with optimized pyrolysis and modification processes. In addition, FM-BC’s extended operational lifespan and reusability in field conditions may translate into lower life-cycle costs compared with conventional sorbents. Strategic alignment with supportive environmental policies, such as subsidies for green materials and clear regulatory frameworks, can facilitate market adoption. Standardized technical guidelines and regulatory clarity will streamline approval processes and encourage broader implementation. By uniting technological scalability (e.g., PRB integration), cost-effectiveness, and policy support, the industrialization of FM-BC can be accelerated—contributing to the development of robust, replicable models for environmental remediation.

In summary, FM-BC holds immense promise as a multifunctional and sustainable material for the synergistic remediation of diverse pollutants in soil and groundwater environments. Through ongoing research and development across materials science, environmental engineering, and policy domains, FM-BC is poised to play a pivotal role in next-generation green remediation technologies.

## Figures and Tables

**Table 1:** Physical and chemical properties of biochar after different iron and manganese loading methods.

Biomass	Preparation Method	Iron Source	Manganese Source	Specific Surface Area/m^2^·g^−1^	Average Pore Size/nm	Iron Mass Fraction/%	Manganese Mass Fraction/%	Oxygen Mass Fraction/%	References
Corn stover	Impregnation pyrolysis	Fe(NO_3_)_3_	KMnO_4_	208.6	2.76	1.11	7.43	6.9	[33]
Loofah	Impregnation pyrolysis	Fe(NO_3_)_3_·9H_2_O	KMnO_4_	187.11	2.91	35.79	22.38	12.29	[47]
Bamboo	Impregnation pyrolysis	FeCl_3_	KMnO_4_	200.88	/	/	/	/	[48]
Wolfsbane straw	Impregnation pyrolysis	Fe(NO_3_)_3_	KMnO_4_	8.80	9.67	/	/	/	[49]
Fava bean straw	Impregnation pyrolysis	FeCl_3_·6H_2_O	KMnO_4_	24.29	14.02	16.0	30.3	51.46	[50]
Waste bone meal	Impregnation pyrolysis	Fe(NO_3_)_3_	KMnO_4_	287.58	6.53	5.42	10.1	39.2	[51]
Algae	Hydrothermal synthesis	FeCl_3_·6H_2_O	MnCl_2_-6H_2_O	180.2	6.11	52.5	14.1	/	[38]
Soybean powder	Hydrothermal synthesis	Iron powder	KMnO_4_	/	/	/	/	/	[52]
Peanut blight	Hydrothermal synthesis	FeCl_3_·6H_2_O	MnCl_2_·4H_2_O	99.05	/	12.96	20.42	/	[53]
Banana leaf	Co-precipitation method	FeSO_4_·7H_2_O	KMnO_4_	187.03	9.18	37.62	12.34	19.12	[54]
Sludge	Co-precipitation	FeCl_3_·6H_2_O	MnCl_2_·5H_2_O	67.34	15.60	/	/	/	[55]
Corn kernel	Co-precipitation method	FeCl_3_·6H_2_O	MnSO_4_·H_2_O	192.41	/	/	/	/	[56]
Pine	Co-precipitation method	FeCl_3_·6H_2_O	MnCl_2_·4H_2_O	280	0.175	9.13	4.85	48.84	[57]
Hickory bushes	Sol-gel method	FeSO_4_·7H_2_O	MnSO_4_, KMnO_4_	/	/	/	/	/	[41]
Corn stover	Sol-gel method	Fe(NO_3_)_3_	Mn(NO_3_)_2_	/	/	/	/	/	[44]
Cotton straw, corn stover, and rice husk	Mechanical ball milling and co-precipitation	FeSO_4_	KMnO_4_	264.48	4.37	/	/	/	[58]
Cotton straw, corn stover, and rice husk	Mechanical ball milling method and co-precipitation method	FeSO_4_	KMnO_4_	226.5–331.5	/	/	/	/	[45]

**Table 2:** Removal of heavy metals by FM-BC.

Heavy Metals	Biomass Raw Material	Preparation Method	Modification Conditions	Reaction Conditions	Adsorption Amount (mg/g)	Adsorption Rate	Removal Mechanism	Reference
As(Ⅲ)	Corn stover	Impregnation pyrolysis	Fe/Mn mass ratio of 1:4, pyrolysis temperature of 600 °C, and N_2_ atmosphere	25 °C, pH = 3	8.39	/	Mesoporous adsorption, oxidation, complexation, and electrostatic interaction	[59]
	Corn stems	Impregnation pyrolysis	Fe/Mn mass ratio of 1:4, pyrolysis temperature of 620 °C, and N_2_ atmosphere	pH = 7	8.25	/	Mesoporous adsorption, oxidation, complexation, and electrostatic interaction	[33]
	Ironwood	Impregnation pyrolysis	Fe/Mn mass ratio of 1:1 and pyrolysis temperature of 800 °C	25 °C, pH = 9	1.89	90.35% of the total amount of the product	Mesoporous adsorption, oxidation, complexation, and electrostatic forces	[60]
As(V)	Pine	Impregnation pyrolysis	Fe/Mn molar ratio of 1:2, pyrolysis temperature of 600 °C, co-precipitation temperature of 80 °C, and N_2_ atmosphere	25 °C, pH = 7.5	3.44	/	Oxidation, complexation, and electrostatic interaction	[57]
Cd(II)	Cotton straw, corn stover, and rice husk	Mechanical ball milling and co-precipitation	Fe/Mn mass ratio of 0.5:3, pyrolysis temperature of 500 °C, and N_2_ atmosphere	25 °C, pH = 5	131.03	96.85%	Complexation, electrostatic interaction, precipitation, and cation-π interaction	[58]
	Rice straw	Impregnation pyrolysis	Fe/Mn molar ratio of 3:5 and pyrolysis temperature of 300 °C	25 °C, pH = 5	120.77	95.20%, pH = 5 120.77	Complexation, electrostatic interaction, precipitation, and cation-π interaction	[61]
	Wolfsbane straw	Impregnation pyrolysis	Fe/Mn mass ratio of 1:4, pyrolysis temperature of 600 °C, and N_2_ atmosphere	25 °C, pH = 5	95.23	/	Complexation, electrostatic interaction, precipitation, and cation-π interaction	[49]
Cr(VI)	Lotus seed	Impregnation pyrolysis	Pyrolysis temperature of 600 °C and N_2_ atmosphere	25 °C, pH = 1.5	21.25	99% of the total amount of the product	Mesoporous adsorption, reduction, complexation, electrostatic interaction, and precipitation	[62]
	Seaweed	Impregnation pyrolysis	Fe/Mn molar ratio of 1:3, pyrolysis temperature of 500 °C, and N_2_ atmosphere	30 °C, pH = 3	104.5	98.90%.	Mesoporous adsorption, reduction, complexation, electrostatic interaction, and precipitation	[63]
	Corn stover	Impregnation pyrolysis	Fe/Mn molar ratio of 1:3, pyrolysis temperature of 400 °C, and N_2_ atmosphere	25 °C, pH = 2	118.03	91.79%.	Mesoporous adsorption, reduction, complexation, and electrostatic interaction	[64]
Cu(II)	Loofah	Impregnation pyrolysis	Using Fe (NO_3_)_3_·9H_2_O and KMnO_4_ impregnation, pyrolysis temperature of 600 °C, and N_2_ atmosphere	25 °C, pH = 5.5	47.64	92.50%	Mesoporous adsorption, complexation, and electrostatic interaction	[47]
	Corn stover	Impregnation pyrolysis	Fe/Mn mass ratio of 1:3, pyrolysis temperature of 600 °C, and N_2_ atmosphere	25 °C, pH = 2.0	64.9	91.79%.	Mesoporous adsorption, complexation, and electrostatic interaction	[34]
	Undaria pinnatifida root	Hydrothermal synthesis	Fe/Mn molar ratio of 2:1 and 453 K (180 °C) hydrothermal for 10 h	25 °C, pH = 5	295.2	/	Mesoporous adsorption and electrostatic interaction	[38]
Hg(II)	Corn stover	Impregnation pyrolysis	Fe/Mn mass ratio of 0.5:3, pyrolysis temperature of 600 °C, and N_2_ atmosphere	25 °C, pH = 7	86.82	72.34%.	Mesoporous adsorption, complexation, electrostatic interaction, and precipitation	[65]
Pb(II)	Rice straw	Impregnation pyrolysis	Fe/Mn molar ratio of 2:5 and pyrolysis temperature of 300 °C	25 °C, pH = 7	165.88	90.42% of the total amount	Mesopore adsorption, complexation, electrostatic interaction, precipitation, and cation-π interaction	[66]
	Corn stover	Co-precipitation method	Pyrolysis temperature of 350 °C and N_2_ atmosphere	25 °C, pH = 5	190.17	/	Mesoporous adsorption, complexation, and electrostatic interaction	[67]
	Corn kernel	Co-precipitation	Pyrolysis temperature of 850 °C and N_2_ atmosphere	25 °C, pH = 5	196.69	/	Mesoporous adsorption, complexation, electrostatic interaction, and precipitation	[56]
Tl(I)	Banana leaf	Co-precipitation	Fe/Mn molar ratio of 2:1 (MnFe_2_O_4_), pyrolysis temperature of 500 °C, and N_2_ atmosphere	25 °C, pH = 6	170.55	99%.	Mesoporous adsorption, oxidation, and complexation	[54]
Zn(II)	Corn stover	Sol-gel method	Pyrolysis temperature of 300 °C	25 °C, pH = 5	/	/	Mesoporous adsorption and complexation	[44]

**Table 3:** Removal of organic pollutants by FM-BC.

Organic Pollutants	Biomass Raw Material	Preparation Method	Modification Conditions	Reaction Conditions	Adsorption Amount (mg/g)	Adsorption Rate (%)	Oxidizing Agent	Removal Mechanism	Reference
Atrazine	Rice straw	Impregnation pyrolysis	Fe/Mn molar ratio of 3:1, pyrolysis temperature of 500 °C, and N_2_ atmosphere	25 °C and pH = 7	/	96.70%	Persulfate	Mesoporous adsorption, oxidation (·OH, SO_4_^−^·, and ^1^O_2_), and complexation	[30]
Ibuprofen	Sawdust	Impregnation pyrolysis	Pyrolysis temperature of 800 °C and N_2_ atmosphere	24 °C and pH = 7	/	95%	Ozone	Mesoporous adsorption, oxidation (·OH and SO_4_^−^·), and hydrogen bonding	[75]
Estrone	Litchi wood	Impregnation pyrolysis	Pyrolysis temperature of 650 °C and N_2_ atmosphere	25 °C and pH = 3	4.18	91.50%	/	Mesoporous adsorption, hydrogen bonding, π-π EDA interaction, and complexation	[76]
Ciprofloxacin	Sludge	Impregnation pyrolysis	Fe/Mn molar ratio of 0.5:1, pyrolysis temperature of 500 °C, and N_2_ atmosphere	25 °C and pH = 5	/	80.85%	/	Mesoporous adsorption, oxidation (·OH and ^1^O_2_), and electrostatic interaction	[77]
Sulfamethoxazole	Corn stover	Co-precipitation	Fe/Mn molar ratio of 2:1, BC pyrolysis temperature of 800 °C, and N_2_ atmosphere	25 °C and 3 ≤ pH ≤ 9	/	92%	Sulfites	Mesoporous adsorption and oxidation (·OH and SO_4_^−^·)	[78]
	Peanut shells	Co-precipitation method	Pyrolysis temperature of 500 °C, and N_2_ atmosphere	25 °C and 3 ≤ pH ≤ 11	/	100%	Peroxymonosulfate	Mesoporous adsorption and oxidation (·OH and ^1^O_2_)	[79]
	Bamboo waste	Impregnation pyrolysis	Fe/Mn molar ratio of 3:2, pyrolysis temperature of 800 °C, and N_2_ atmosphere	25 °C and pH ≈ 5.6	/	97.90%	Peroxymonosulfate	Oxidation (·OH and ^1^O_2_), electrostatic interaction, hydrogen bonding, and π-π EDA effect	[80]
	Sludge	Co-precipitation	Pyrolysis temperature of 600 °C and N_2_ atmosphere	25 °C and 3 ≤ pH ≤ 11	/	98.80%	Persulfate	Mesoporous adsorption, oxidation (·OH and ^1^O_2_), and hydrogen bonding	[81]
Sulfamethoxazole	Rice straw	Hydrothermal synthesis	Pyrolysis temperature of 500 °C	25 °C and natural pH	/	83.80%	/	Mesoporous adsorption and oxidation (·OH)	[82]
Activated Blue 19	Sludge	Impregnation pyrolysis	Fe/Mn molar ratio 1:1 and pyrolysis temperature of 600 °C	25 °C and 3 ≤ pH ≤ 9	/	98.33%	Persulfate	Mesoporous adsorption, oxidation (·OH), and complexation	[83]
Carbamazepine	Soybean powder	Hydrothermal synthesis	Fe powder 0.17 mol/L + KMnO_4_, pyrolysis temperature of 600 °C, and N_2_ atmosphere	25 °C and pH ≈ 7	/	99%	Peroxymonosulfate	Oxidation (·OH and ^1^O_2_), π-π EDA effect, and hydrogen bonding effect	[52]
Dibutyl phthalate, Bis(2-ethylhexyl) phthalate	Corn stover	Impregnation pyrolysis	Fe/Mn mass ratio of 1:6, pyrolysis temperature of 600 °C, and N_2_ atmosphere	Room temperature	/	/	/	Mesoporous adsorption and electrostatic interaction	[84]
	Corn stover	Impregnation pyrolysis	Pyrolysis temperature of 600 °C	Natural temperature and natural pH	/	/	/	Mesoporous adsorption, electrostatic interaction, and complexation	[85]
Rhodamine B	Straw	Sol-gel method	Fe/Mn molar ratio of 2:1 and pyrolysis temperature of 300 °C	Room temperature and pH = 7	/	100%	Potassium persulfate	Oxidation (·OH and SO_4_^−^·), π-π EDA action, and complexation	[86]
Thiacloprid	Sludge	Co-precipitation method	Pyrolysis temperature of 600 °C and N_2_ atmosphere	25 °C and 3 ≤ pH ≤ 11	/	94.10%	Periodate	Mesoporous adsorption and oxidation (·OH and IO_3_·)	[87]
Thiamethoxam	Straw	Sol-gel method	Fe/Mn molar ratio of 2:1 and pyrolysis temperature of 600 °C	Room temperature and natural pH	/	99%	Potassium persulfate	Mesoporous adsorption, oxidation (·OH and SO_4_^−^·), and surface complexation	[88]
Bisphenol A	Straw	Impregnation pyrolysis	Pyrolysis temperature of 800 °C and N_2_ atmosphere	20 °C and 3 ≤ pH ≤ 10	/	100%	Peroxymonosulfate	Mesoporous adsorption and oxidation (·OH, SO_4_^−^·, and ^1^O_2_)	[89]
Tetracycline	Rice straw	Hydrothermal synthesis	Pyrolysis temperature 600 °C and N_2_ atmosphere	25 °C and 5 ≤ pH ≤ 9	/	85%	Peroxymonosulfate	Mesoporous adsorption and oxidation (·OH and ^1^O_2_)	[90]
	Platycodon grandiflorum twigs	Impregnation pyrolysis	Pyrolysis temperature of 800 °C and N_2_ atmosphere	25 °C and 2.29 ≤ pH ≤ 11.43	/	97.90%	Peroxymonosulfate	Oxidation (·OH and ^1^O_2_), electrostatic interaction, hydrogen bonding, and π-π EDA interaction	[91]
Acid red 88	Cedar sawdust	Impregnation pyrolysis	Fe/Mn mass ratio of 1:1, microwave radiation power of 200 W, and N_2_ atmosphere	25 °C	/	98.84%	Persulfate	Mesoporous adsorption and oxidation (·OH and SO_4_^−^·)	[92]
Methylene blue	Alder	Impregnation pyrolysis	Fe/Mn molar ratio of 2:1, pyrolysis temperature of 800 °C, and N_2_ atmosphere	25 °C and 3 ≤ pH ≤ 10	97.41	97.41	/	Mesoporous adsorption, complexation, hydrogen bonding, and π-π EDA effect	[93]
Anaerobic sludge	Peanut shells	Hydrothermal synthesis	Pyrolysis temperature 800 °C and N_2_ atmosphere	37 °C and natural pH	/	/	//	Mesoporous adsorption and oxidation	[53]
Levofloxacin	Wine lees waste	Impregnation pyrolysis method and co-precipitation method	Pyrolysis temperature of 800 °C and N_2_ atmosphere	25 °C and pH = 5	181	91.50%	/	Mesoporous adsorption, hydrogen bonding, π-π EDA effect, and salinization effect	[94]

**Table 4:** Removal of phosphate and nitrate by FM-BC.

Phosphate/Nitrate	Biomass Raw Material	Preparation Method	Modification Conditions	Optimal Reaction Conditions	Maximum Adsorption Amount (mg/g)	Maximum Adsorption Rate	Removal Mechanism	Reference
Phosphate	Cotton straw, corn stover, and rice husk	Impregnation pyrolysis and mechanical ball milling	Fe/Mn coating (ball milling for 6 h) and N_2_ atmosphere	24.85 °C and pH = 3	53.3	94.72%.	Mesoporous adsorption, complexation, electrostatic interaction, and precipitation	[45]
	Microalgae	Impregnation pyrolysis	Fe/biomass = 1.25 (*w*/*w*), Mn/biomass = 1.10 (*w*/*w*), and pyrolysis temperature of 650 °C, N_2_ atmosphere, and EDTA chelation	25 °C and pH = 7	23.23	91.60%	Mesoporous adsorption, complexation, electrostatic interaction, and precipitation	[97]
	Rice straw	Impregnation method	Fe/Mn molar ratio of 3:1	Room Temperature and pH = 6	135.88	-	Complexation, electrostatic interaction, and precipitation	[98]
	Fruit shell (apricot shell)	Impregnation method	Fe/Mn molar ratio of 1:1 and drying temperature of 378 K	25 °C and 4 ≤ pH ≤ 10	4.69 (under 10 mg/L phosphorus concentration)	93.24%	Complexation and electrostatic interaction	[99]
Nitrate	Coconut shell	Microelectrolysis	Shell iron/biochar/manganese sand = 6:2:1 (mass ratio) to construct a microelectrolysis system	27 °C and pH = 7	/	80.30%	Microelectrolysis and complexation	[100]
	Wheat straw	Impregnation pyrolysis	Fe/Mn molar ratio of 1:1, pyrolysis temperature of 400 °C, and N_2_ atmosphere	25 °C and 1 ≤ pH ≤ 9	37.36	78.70%	Mesoporous adsorption, complexation, and electrostatic interaction	[101]

## Data Availability

No new data were created for this review paper.
